# Continuous In‐Situ Water Stable Isotopes Reveal Rapid Changes in Root Water Uptake by *Fagus sylvatica* During Severe Drought

**DOI:** 10.1111/pce.70055

**Published:** 2025-07-10

**Authors:** Laura Kinzinger, Simon Haberstroh, Judith Mach, Markus Weiler, Natalie Orlowski, Christiane Werner

**Affiliations:** ^1^ Ecosystem Physiology, Faculty of Environment and Natural Resources University of Freiburg Freiburg im Breisgau Germany; ^2^ Hydrology, Faculty of Environment and Natural Resources University of Freiburg Freiburg im Breisgau Germany; ^3^ Institute of Soil Science and Site Ecology Chair of Forest Sites and Hydrology, TU Dresden Germany

**Keywords:** drought, *Fagus sylvatica*, precipitation, root water uptake, water stable isotopes

## Abstract

Adaptation of root water uptake (RWU) is critical for drought resilience in temperate forest trees, yet information on water sources and uptake depths dynamics is scarce. Continuous in‐situ stable isotope measurements in soil and xylem water of *Fagus sylvatica* during the severe drought 2022 revealed daily changes in RWU depth and water ages. Xylem water comprised mainly recent precipitation in early summer, but winter and spring precipitation contributed up to 70% during drought, with longer transit times (206 ± 60 days) compared to summer precipitation (62 ± 11 days). Concurrently, trees shifted RWU to deeper soil layers while also responding to individual precipitation events by absorbing fresh precipitation from topsoil layers within 2–4 days, demonstrating the significance of individual precipitation events for tree water dynamic. *F. sylvatica* used > 80% of a fresh precipitation event before drought, but < 20% during recovery, indicating potential drought legacies on precipitation use. Unravelling these rapid dynamics in RWU and water ages offers novel insights into the importance of single and seasonal precipitation events for forest water fluxes.

## Summary statement

- Continuous in‐situ measurements of water stable isotopes reveal that *F. sylvatica* adapts its water uptake depth within few days to water availability during drought leading to significant xylem water age changes.
- Drought further alters the velocity of the uptake of single precipitation events.

## 1 Introduction

In light of current and predicted climate change, research has increasingly focused on gaining a better understanding of the ecophysiological responses and resilience of trees to changing environmental conditions, such as the series of recurrent hot drought events in Central Europe in recent years (Knutzen et al. 2025; Schuldt et al. 2020; Rukh et al. 2023). Ecophysiologically, trees can increase their drought resilience in various ways, such as by reducing stomatal conductance to reduce transpirational water loss (Granier et al. 2007; Hölscher et al. 2005). While such aboveground responses are well studied and understood, belowground processes, such as dynamic changes in root water uptake (RWU) depths and tree water ages in response to precipitation during drought are less well understood (Brinkmann et al. 2018; Floriancic et al. 2024).

Seasonally, trees shift their RWU to deeper soil layers in response to water limitations, for example, by the means of deep tap roots (Bachofen et al. 2024; Kühnhammer et al. 2023; Brinkmann et al. 2019; Dawson et al. 1996; Grossiord et al. 2017; Zapater et al. 2013), which is driven by layer specific soil moisture dynamics (Plamboeck et al. 1999; Nehemy et al. 2021; Bachofen et al. 2024). Recent studies mostly indicate that trees in Central Europe mainly use precipitation fallen in winter, while fresh precipitation contributed only to a minor fraction to total water use in summer (Goldsmith et al. 2022; Floriancic et al. 2024). However, little is known about the dynamic use of seasonal water sources, for example, winter precipitation, which is stored in the soil versus fresh precipitation water (Brinkmann et al. 2018; Allen et al. 2019; Sprenger et al. 2019; Goldsmith et al. 2022; Floriancic et al. 2024). Brinkmann et al. (2018) found precipitation fallen during the growing season was equally important to antecedent winter precipitation for temperate trees, such as for example *Fagus sylvatica* or *Picea abies*. In contrast, Gessler et al. (2022) indicated that *F. sylvatica* took up mainly recent precipitation from top soil layers during the severe drought 2018. Thus, even single precipitation events during the vegetation period, for example, during or after long periods of drought, could be of high importance for temperate trees to sustain transpiration. However, uptake of fresh precipitation water might differ before and after long drought periods, for example, due to drought related plasticity of fine roots in the topsoil (e.g., Mainiero and Kazda 2006; Meier and Leuschner 2008). Understanding the role of winter and summer rainfalls for forests and the dynamic changes could be of particular importance with respect to anticipated changes in precipitation pattern in Central Europe with progressing climate change (IPCC 2023).

The main limitation in assessing such high temporal dynamics in RWU depths and xylem water ages to answer the above postulated questions has been the methodological restriction of destructive and labour intense measurement methods. Tree water sources have traditionally been estimated using stable isotopes of water (^2^H and ^18^O) (Amin et al. 2020; Brinkmann et al. 2019; Dawson et al. 1996; Dawson and Ehleringer 1991; Dubbert and Werner 2018; Kahmen et al. 2022) with methods, such as the cryogenic vacuum extraction (CVE) (Orlowski et al. 2018; Haberstroh et al. 2024). The labour‐intensive sampling for CVE or other destructive methods generally leads to limited data (e.g., monthly values), which restricts high temporal resolution of water uptake and age dynamics (Allen et al. 2019; Brinkmann et al. 2018). Thus, we might miss important changes in RWU or water ages used for transpiration, which might occur on a (sub‐)daily basis, for example, as a response to single precipitation events. However, short‐term responses, especially during drought might be important in explaining species‐specific drought resilience. Different species do show different plasticity in RWU (e.g., Schwinning et al. 2002; Liu et al. 2023), and thus, a dynamic, species‐specific response to fresh precipitation is to be expected. After a drought experiment, mature *F. sylvatica* trees showed a rapid physiological recovery within a day after irrigation (Hesse et al. 2023). Similarly, Kahmen et al. (2021) found an immediate uptake of infiltrating mobile soil water by broadleaved species during a pulse labelling experiment. These results suggest that *F. sylvatica* responds rapidly to individual precipitation events, raising questions about the importance of such events, particularly during or after periods of drought. However, recent studies were confined to short time series during single experiments and thus, insights on short‐term dynamics of RWU and xylem water ages as modulated by drought or seasonal water availability are still scarce.

Significant advances in measurements with field‐deployable laser spectroscopy and in‐situ membrane‐based probes now provide a much higher temporal resolution (daily to sub‐daily) than destructive sampling and are able to capture the isotopic signature of soil and tree xylem by using the same measurement system (Kübert et al. 2023; Kühnhammer et al. 2023; Mennekes et al. 2021; Seeger and Weiler 2021; Volkmann et al. 2016; Werner et al. 2021). These methods allow for frequent measurements, enabling an investigation of rapid changes in xylem water isotopic composition in response to environmental conditions, such as precipitation events and droughts (Volkmann et al. 2016; Seeger and Weiler 2021). Using novel in‐situ measurements can further improve modelling of ecohydrological fluxes for example, by isotopic mixing models (Beyer et al. 2020; Kinzinger et al. 2023). Thereby, models can estimate the dynamic use of water pools on a daily basis, filling the gap of urgently needed pool‐weighted approaches (Dubbert et al. 2019). By integrating isotopic data with other measurements (e.g., soil moisture, sap flow), a better understanding of the influence of environmental condition on tree water source variation also allows a more robust seasonal evaluation of water transit times and xylem water age.

Analyzing the naturally occurring isotopic composition of water in trees and soils allows observations of long‐term water use patterns. Thereby, we often fail to accurately determine the sources on which plants depend or are not able to consider all potential sources (von Freyberg et al. 2020). This is primarily due to uncertainties associated with the potential tree water sources and the inherent assumptions in the mixing model concept. As a result, models based on natural abundance of water stable isotopes may overestimate deep‐layer soil water use by trees (Wang et al. 2023). Applying an deuterium‐enriched irrigation can increase the isotopic differences between the tree source water and helps to better discriminate between different water sources used by trees (e.g., Kahmen et al. 2021; Magh et al. 2020; Schwinning et al. 2002). Thus, combining continuous in‐situ isotopic measurements with isotopic labelling as well as long‐term monitoring can help to gain a new understanding of rapid changes in plant response to drought stress including changes in RWU (Beyer et al. 2020; Dubbert et al. 2023; Orlowski et al. 2023). First studies using in‐situ isotope measurements were able to detect species‐ and precipitation‐dependent plant responses to single precipitation events. For example, Kühnhammer et al. (2023) showed a rapid response within 2 days of some tropical trees to a strong deep label pulse (> 18 mm) after an experimental drought, while other species in the same experiment showed delayed responses (> 55 days) (Werner et al. 2021). Ring et al. (2024) found an uptake of recent precipitation in urban trees during drought only for larger precipitation events (> 18 mm), while smaller precipitation amounts were not detectable in tree xylem water during the 2022 summer drought. These results highlight the fact that the dynamics of RWU are influenced by external and species‐dependent factors, which must be considered to better understand drought resilience.

Here, we combined daily isotopic measurements (δ^2^H and δ^18^O) of tree xylem water of *F. sylvatica*, soil water at different depths and precipitation with soil moisture and tree water flux measurements. We used natural abundance measurements for a long‐term observation over an entire vegetation period of 2022, which included a severe drought. Furthermore, we applied δ^2^H pulse labelling during wet and dry conditions for a more detailed analysis of short‐term tree responses and to compare natural abundance response with those of pulse labelling experiments. Our main objective was to investigate the RWU of *F. sylvatica* on a daily resolution to detect potential changes in RWU depth and xylem water ages as a response to single precipitation events and changes of precipitation water transit times before, during and after the severe drought. We hypothesise that (1) *F. sylvatica* dynamically shifts its root water uptake depth in response to soil water availability and precipitation events, thereby, significantly affecting the age of water used for transpiration and (2) that therefore uptake of recent precipitation water by *F. sylvatica* differs during pre‐drought, drought and post‐drought condition.

## 2 Materials and Methods

### 2.1 Field Site and Environmental Data

The field site is located in the Black Forest in SW‐Germany (N48.2562°, E7.9220°). Measurements took place in a forest dominated by *Fagus sylvatica L*., in mixture with *Picea abies* L., *Quercus petraea* L., *Pseudotsuga menziesii* (MIRBEL) and other species, on silty and loamy soils with a groundwater level > 2 m depth (see also Kinzinger et al. 2023). Local meteorological conditions were measured in a clearing 600 m from the field site to allow for precipitation measurements unaffected by tree crowns. Air temperature (CS215 sensor; Campbell Scientific, Shepshed, UK), precipitation (Rain Collector II; Davis Instruments, Hayward, CA, USA), and photosynthetic photon flux density (PPFD) (LI‐190 Terrestrial Quantum Sensor; LI‐COR Biosciences, Lincoln, NE, USA) were recorded at 5‐min intervals on an CR1000 data logger (Campbell Scientific Ltd., Shepshed, UK). Data were compared with the long‐term average recorded by the German Weather Service (DWD 2022) at Elzach‐Fisnacht, approx. 15 km distance to the field site.

### 2.2 Experimental Design

The experimental design included nine selected *F. sylvatica* individuals (*n* = 9) between 20 and 29 m height, standing in groups of three. Direct neighbouring trees of the selected individuals were only *F. sylvatica* trees. Volumetric soil water content (VWC) was measured continuously in three soil profiles at four depths (0.05, 0.20, 0.40, 0.90 m) in the centre of each forest patch using SMT100 sensors (TRUEBNER GmbH, Neustadt, Germany), connected to CR1000 data loggers (Campbell Scientific Ltd., Shepshed, UK).

Sap flux density (*J*_*S*_) was calculated from sap flow velocities measured by heat pulse velocity (HPV) sensors with three 30 mm long, 1.3 mm diameter stainless steel needles (HPV‐06, Implexx, Melbourne, VIC, Australia) installed at breast height (1.3 m). The thermistors at 10‐ and 20‐mm depth below the bark along the needles measured with an accuracy of ± 0.2°C with a resolution of 0.001°C (Forster 2021). Measurements were recorded on a CR1000 data logger (Campbell Scientific Ltd, Shepshed, UK) in 10‐min intervals. Sap wood depth was determined by the light transmission method using illuminated tree cores to obtain an estimate of the active water transport zone (Munster‐Swendsen 1987, Børja et al. 2016, Quiñonez‐Piñón and Valeo 2018). Sap flow was calculated using a combination of the heat ratio method (HRM) and the Tmax method (Dual Method Approach) as described by Forster (2019; 2021). Needle misalignment was corrected using a zero‐flow night correction, where nocturnal sap flow was defined as zero (2 AM and 4 AM) with VPD < 0.01 kPa and PAR > 5 μmol m^−^^2^ s^−^^1^ and the distance between the upper and lower needles was corrected. The sap wood area per ground area of *F. sylvatica* trees was estimated using the sap wood depth measured at the site and the forest floor area. Transpiration rates represent stand scale transpiration and were calculated based on measured sap flow data and sap wood area per m^2^ forest floor.

### 2.3 In‐Situ Isotopic Measurement System

δ^2^H and δ^18^O values were measured continuously in the tree xylem and soil water using probes with a diffusion‐dilution sampling design (Kinzinger et al. 2023; Volkmann and Weiler 2014) that transported air and water vapour to a Cavity Ring‐Down Spectroscopy (CRDS) water isotope analyser (L2130‐*i*, Picarro, Santa Clara, USA) in the field. Probes were installed at four depths in each of the three soil profiles (0.05, 0.20, 0.40 and 0.90 m) and in the xylem of nine beech trees at their stem base. Water stable isotope values (δ^2^H and δ^18^O) were expressed relative to the Vienna Standard Mean Ocean Water (VSMOW2) in per mill (‰) (Gonfiantini 1978). Isotope measurements had an accuracy of ± 1‰ for δ^18^O and ± 2‰ for δ^2^H. The CRDS has a precision of 0.025‰ for δ^18^O and ± 0.1‰ for δ^2^H in vapour mode (CRDS, L2130*‐i*, Picarro, Santa Clara, USA). Three air‐tight containers filled with different in‐house isotope standards (δ^18^O = − 24.44‰ ± 0.29‰; −17.98‰ ± 0.29‰; −14.80‰ ± 0.29‰ and δ^2^H = −96.12‰ ± 0.82‰; −60.40‰ ± 0.78‰; −0.62‰ ± 0.88‰) calibrated against VSMOW2 were used continuously for drift correction. The CRDS was calibrated using in‐house water isotope standards and data were then corrected for the water concentration (cf. Haberstroh et al. 2024). Herbstritt et al. (2024) showed, that in‐situ measurements are very robust with regard to organic contamination and do not influence the final results. Additionally, organic contamination was checked using the CH_4_ measurement parameter recorded by the analyser. Later, a liquid‐gas correction was applied using a linear regression between liquid and gas measurement values for the three in‐house water isotope standards to convert measured xylem and soil water vapour values, accordingly. Each probe was measured daily to bi‐daily. For a detailed description of the probe functioning and data correction see also Kinzinger et al. (2023).

Additionally, isotope values of precipitation were measured by collecting precipitation water using Rain Collectors II (Davis Instruments, Hayward, CA, USA) at the local meteorological station (*n* = 1) and as throughfall using 9 m long V‐shaped aluminium channels with an area of 4200 cm^2^ draining into rain collectors placed at the forest site (*n* = 3). Samples were collected monthly in winter and weekly in summer between November 2021 and November 2022 in 100 mL amber glass bottles with no headspace, tightly sealed and stored at 4°C to prevent fractionation due to evaporation. Precipitation water was analysed in the laboratory using a water isotope analyser (L2130‐*i*, Picarro, Santa Clara, USA) equipped with an A0211 autosampler and a V1102‐i vaporisation module operated at 120°C. Data were calibrated and drift corrected using three in‐house isotope standards (δ^18^O = −14.86‰ ± 0.16‰; −9.47‰ ± 0.16‰; 0.30‰ ± 0.16‰ and δ^2^H = −107.96‰ ± 0.6‰; −66.07‰ ± 0.6‰; 1.53‰ ± 0.6‰, respectively).

### 2.4 Δ^2^H Pulse Labelling Experiments

In addition to naturally occurring precipitation events, two isotope labelling experiments with deuterium enriched water were conducted to verify the RWU patterns observed under natural abundance precipitation. Labelling experiments were carried out twice during summer 2022. Each time, 160 m^2^ of each *F. sylvatica* patch was irrigated evenly. The first δ^2^H labelling pulse was applied with 7 mm irrigation (intensity: 252 mm h^–1^) on 10/06/2022 after several days of naturally occurring rainfall. The second δ^2^H labelling pulse was applied during the 2022 severe drought period on 02/08/2022 with an irrigation amount of 23 mm (intensity: 126 mm h^–1^). Irrigated water was labelled with + 1017.08‰ ± 21.24‰ δ^2^H and + 801.15‰ ± 23.82‰ δ^2^H, respectively. Water was applied manually using water sprinklers connected to a deuterium enriched water reservoir of 45,000 L. Direct watering of tree trunks was avoided to minimise stem flow generation.

### 2.5 Root Water Age and Water Uptake Depth Modelling

Estimation of the contribution of each precipitation event to tree RWU was based on a Bayesian isotope mixing model (R package “MixSIAR”) (Stock and Semmens 2016; Stock et al. 2018), using δ^2^H and δ^18^O values of tree xylem and precipitation water. Water from different precipitation events was defined as a possible water source for xylem water. Precipitation events were weighted by two factors: (1) amount of precipitation water divided by (2) time elapsed since precipitation had occurred. The model was run for each day with isotope measurements with the Markov chain Monte Carlo function, considering residual and process errors (residual * process error structure). The age of multiple day precipitation events was calculated using the day of first precipitation. As the age of each precipitation event was known, the age distribution of RWU and soil water could be determined using a cumulative age distribution curve, displaying the contribution of different water ages to total RWU.

A second MixSIAR approach using the daily δ^2^H and δ^18^O values of water in the four soil depths as possible sources for daily measured xylem δ^2^H values was used to determine the RWU depth per day and its dynamics. The soil depths were weighted according to the root density distribution. Root density at the site was measured in the upper 0.40 m by taking three 1000 cm^3^ soil cores per depth and extracting and weighing root material. Thereby, the relative root distribution in the upper 0.40 m was found to be 28%, 30% and 42% at 0.05, 0.20 and 0.40 m depth, respectively. Other studies have described the root distribution of *F. sylvatica* as being concentrated in the upper 0.70 m (Brinkmann et al. 2019; Meißner et al. 2012). As root distribution was not determined below 0.40 m depth, an estimated 1% of the total root density was assumed to be in 0.90 m soil depth to minimise but not dismiss the likelihood of water uptake from deep soil layers. By using the four measurement depths as reference points a mean RWU depth was calculated by weighing the specific depths by their modelled RWU contribution (%).

To compare different periods, 2022 was divided into an initial wet, dry down, dry, recovery and final wet period, based on the VWC and frequency of precipitation. During the initial wet period, precipitation occurred weekly and the average soil VWC was 19.3% ± 3.5% with mean transpiration rates of 0.99 ± 0.42 mm d^–1^. During the dry‐down period, VWC showed a declining trend from 18.5% to 12.2% and remained below 13.0% during the subsequent drought. During these periods, precipitation events with < 10 mm were less frequent and transpiration rate declined during drought to a minimum of 0.19 mm d^–1^. The period after the second δ^2^H labelling pulse during the drought was separated in the statistical analysis from the previous dry period. The recovery period was defined by the onset of weekly occurring precipitation with > 10 mm and an increase in soil VWC from 9.9% to 17.3%. Mean transpiration rates remained low with 0.28 ± 0.17 mm d^–1^. The final wet period was defined by an average soil VWC of 17.2% ± 1.4% and a mean transpiration rate of 0.19 ± 0.13 mm d^–1^. Significant differences in mean RWU depth as well as differences in the cumulative water age between the different periods were determined by a linear regression. The correlation between VPD (kPa) and transpiration rate (mm d^–1^) was best described by a logarithmic regression.

The transit time of individual precipitation events was calculated by evaluating the time during which a precipitation contributed at least 10% to the RWU depth. In addition, we investigated if the use of individual precipitation events (*uptake of precipitation events*) differed depending on the environmental conditions (e.g., wet vs. dry) in the five defined periods. To determine the trees' absolute water uptake and uptake rate from single precipitation events, we fitted an asymptotic regression (using the “drm()” function in R) (Ritz et al. 2015):

(1) $$\mathrm{Uptake}\;\mathrm{of}\;\mathrm{precipitation}\;\mathrm{events}=a-(a-b)e^{\left(-c\;t\right)},$$

where *a* is the maximum percentage a tree can use from a precipitation event, *b* is the used precipitation amount at Day 0 (i.e., the day of the precipitation event) and *c* is proportional to the relative rate of use of precipitation increase while Day increases (i.e., the speed of precipitation water uptake). Regressions were tested using the DHARMa package (Hartig 2022) including Kolmogorow‐Smirnow test, dispersion and outlier test. Significant differences between cumulative age distribution curves and asymptotic curves of precipitation water uptake were determined by using linear model, which were also tested using the DHARMa package (Hartig 2022).

## 3 Results

### 3.1 Meteorological Conditions and Transpiration Rates in 2022

**Figure 1:**
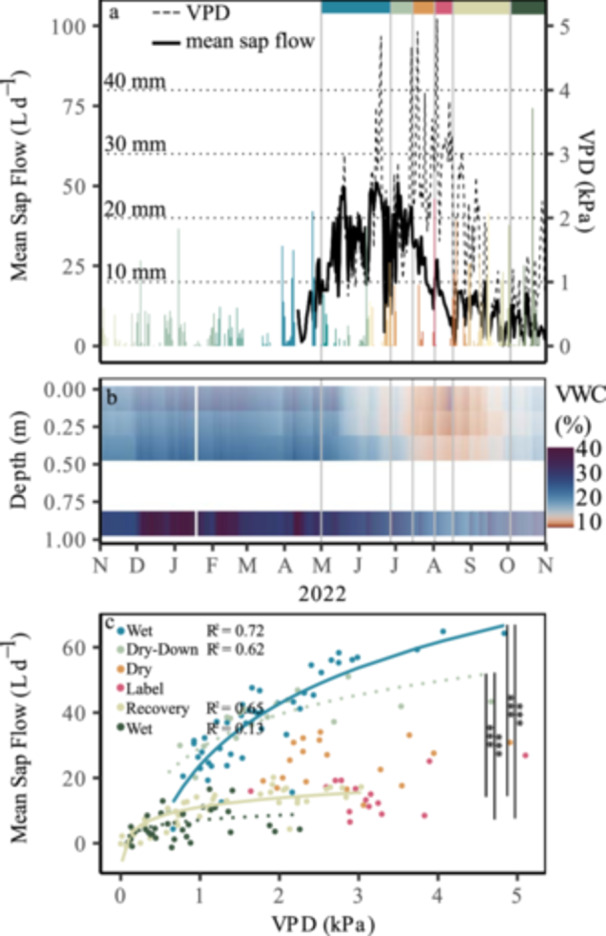
Meteorological data of 2022 including precipitation (mm; coloured by period), vapour pressure deficit (VPD) (kPa), transpiration (mm d^–1^) (a) and soil volumetric water content (VWC) in four soil layers (b) during the five periods (wet, dry‐down, dry, recovery, wet) defined in 2022. (c) shows the significant changes of the logarithmic regression of Mean Sap Flow (L d^–1^) with increasing VPD (kPa) between the different periods.

The year was characterised by above average temperatures and a lack of precipitation during summer 2022 in central and southern Europe, which did also affect our study site. High daily maximum VPD > 4.8 kPa (Figure 1a) and high air temperature with maximum temperatures of > 35°C during single days in June, July and August were recorded. The mean air temperature during the summer months May, June, July and August was 2°C–3°C above the long‐term average (1991–2020) of 13.6°C–19.0°C at the DWD station Elzach‐Fisnacht. Precipitation in July 2022 was 85% lower than the long‐term average. In June and August 2022 precipitation was reduced by 23% and 16%, respectively, compared to the period from 1990 to 2020. Consequently, a strong decline in soil VWC was observed from July onwards leading to a VWC of less than 10% in the upper 0.40 m in August. We therefore defined the period from July to mid‐August as a drought period. Due to frequent precipitation from mid‐August onwards, soil VWC increased. However, soil water resources were still not replenished in October (18% VWC in October after re‐wetting) compared to pre‐drought levels (22%–25% VWC pre‐drought), highlighting the severity of the 2022 drought (Figure 1b).

Sap flow rates (Figure 1a) showed high values (64.8 ± 36.6 L d^–1^) during the early summer 2022 and a clear downregulation with the onset of drought to a minimum of 8.5 ± 5.1 L d^–1^ in mid‐August. Although VWC increased in October, sap flow rates remained low from mid‐August to mid‐September (20.2 ± 11.8 L d^–1^) and did not recover to pre‐drought levels in 2022.

A logarithmic regression between sap flow (L d^–1^) and VPD (kPa) demonstrated a weaker increase of sap flow with increasing VPD in the recovery period compared to the initial wet period (*p* < 0.001; Figure 1c). These results highlight that the response of sap flow to VPD in the recovery period differed strongly from the response in the pre‐drought period.

### 3.2 Isotopic Composition of Precipitation, Soil and Xylem Water

**Figure 2:**
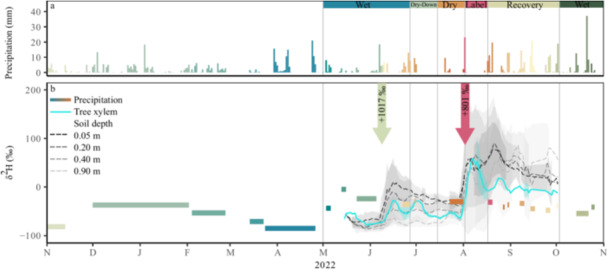
Precipitation amount (a) and daily isotopic values (b) in tree xylem water (blue line) and soil water in four depths (0.05, 0.20, 0.40 and 0.90 m; grey to black dashed lines). Lines illustrate the 5‐day running mean in tree xylem water and soil water in four depths (0.05, 0.20, 0.40 and 0.90 m). Labelling events with δ^2^H values are depicted by arrows in panel (b). [Color figure can be viewed at wileyonlinelibrary.com]

Natural abundance isotopic values of precipitation varied in 2022 from −84.91‰ to −4.87‰ (δ^2^H) and −11.74‰ to 1.74‰ (δ^18^O) (δ^2^H pulse labelling excluded), with most depleted values in November and April and most enriched values in May (Figure 2). During the severe drought, two δ^2^H labelling pulse experiments were conducted (10/06/2022 and 02/08/2022). Those were clearly distinguishable from natural precipitation (Figure 2b).

The first labelling pulse of 7 mm and a δ^2^H value of + 1017.08‰ was conducted during rather moist conditions. It caused a δ^2^H enrichment in the tree xylem of + 58.0 ± 29.9‰. In the soil, the pulse labelling showed only weak infiltration of labelled water into deeper soil layers. The enrichment in the soil decreased clearly with increasing depth (Figure 2b; 0.20 m = + 125.4‰ ± 20.0‰, 0.40 m = +98.6‰ ± 26.6‰, 0.90 m = + 31.9‰ ± 8.2‰). The second, larger δ^2^H labelling pulse of 23 mm and a δ^2^H value of + 801.15‰ was conducted during the peak of the drought (August 2022). It caused a strong increase in δ^2^H values within the tree xylem of 152.0‰ ± 53.4‰. In the soil, we found the highest enrichment in 0.20 m depth (with δ^2^H of 116.5‰ ± 124.8‰), followed 0.05 m and 0.40 m depth (69.5‰ ± 61.0‰ and 77.6‰ ± 66.1‰, respectively) and in 0.90 m depth (31.1‰ ± 22.6‰).

We also found secondary peaks of enrichment in the trees' xylem water after the δ^2^H labelling pulses during strong natural precipitation events, for example, 2 weeks after the second pulse labelling (17/08/2022). Similar δ^2^H enrichment occurred three and 6 weeks after the first pulse labelling, correlating with increases in sap flux (Figure 1).

### 3.3 Dynamic Shifts in Root Water Uptake Depths During the Dry Summer 2022

Using daily δ^2^H values of tree xylem and soil water, we were able to model the RWU depth and source of trees with MixSIAR on 125 out of 194 days of the growing season Missing days were linearly interpolated. RWU depth estimated by the MixSIAR model changed significantly over the course of the summer drought 2022. During the initial wet period, the model predicted trees water uptake mainly from 0.40 m (on average 60%), followed by 0.20 m (23%), 0.05 m (11%) and 0.90 m (7%).

During drought, we found deep soil water uptake to play an increasingly important role in the trees' water supply. RWU from deep soil layers (represented with a reference measurement in 0.90 m depth) was relatively small with < 13% but increased significantly during drought to 34% (*p* < 0.05).

**Figure 3:**
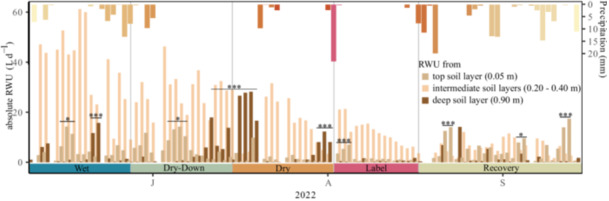
Mean absolute RWU depth (L d^−^^1^) during the five periods in 2022 based on relative RWU contribution of the four measurement points in the soil profile (0.05, 0.20, 0.40 and 0.90 m) and daily transpiration rates (mm d^−1^). Significant increases in RWU from top soil layer (light brown or deep soil layers (dark brown) are marked with asterisks. [Color figure can be viewed at wileyonlinelibrary.com]

Our results further highlight the ability of trees to rapidly shift RWU depth to top soil layers for uptake of recent precipitation water. Most of the water found in tree xylem was taken up from 0.40 m depth during the vegetation period ( > 49%). However, we found a significant shift in absolute RWU from deep soil layers (0.90 m) after seven or more days without precipitation in June and July (*p* < 0.001). Within these periods, VWC in shallow soil layers (0.05−0.40 m) decreased from 14.0% to 12.3% in June and from 15.0% to 9.2% and from 9.0% to 8.4% in July. Whenever individual precipitation events occurred during these dry‐down periods, we observed a shift in absolute RWU to topsoil layers (0.05 m; *p* < 0.05) within two to 4 days in June, July, after the pulse labelling in August and in September (Figure 3).

### 3.4 Plant Response to Two Different Pulse Labelling Experiments

To understand the changes in tree water ages and their dynamics during the vegetation period, we used the MixSIAR RWU model to estimate xylem water compositions. The MixSIAR model predicted the most likely contributions to RWU for each individual precipitation event for each tree observed. Thereby, the model output always introduced an uncertainty expressed as the standard deviation of the predicted contribution, which did not differ strongly between the individual trees (0.066 ± 0.010) or the natural abundance precipitation events (0.089 ± 0.045). The standard deviations of the 7 and 23 mm δ^2^H pulse labelling experiments were 0.006 and 0.014, respectively, indicating a slightly lower uncertainty compared to natural precipitation events.

**Figure 4:**
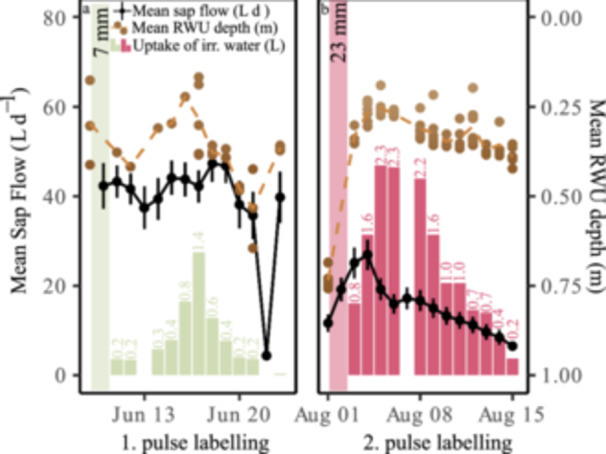
Dynamics in mean RWU depth, sap flow, and uptake of irrigated water simulated by a pulse labelling on 10 June 2022 during wet conditions (a) and on 02 August 2022 during drought (b). [Color figure can be viewed at wileyonlinelibrary.com]

A comparison of the pulse labelling experiments showed a faster response time following the irrigation during drought compare to the irrigation during moister conditions. The peak uptake of irrigated water, averaging 1.4 L, was observed 7 days after the first pulse labelling of 7 mm (Figure 4a). In contrast, just 3 days after the second pulse labelling of 23 mm during drought, the highest uptake of irrigated water was recorded, averaging 2.3 L (Figure 4b), depicting differences in uptake velocity. During the initial moist period, no clear shifts in mean RWU depth (calculated as a weighted mean of measurements within the top metre of soil) were observed with a mean RWU depth of 0.36 ± 0.05 m. In contrast, during drought conditions, *F. sylvatica* altered its mean RWU depth towards top soil layers, and thus, fresh precipitation water. Within the first 3 days, the RWU depth decreasing from 0.73 ± 0.03 to 0.26 ± 0.03 m, before gradually declining over the subsequent 2 weeks.

### 3.5 Seasonal Contribution and Water Transit Times

**Figure 5:**
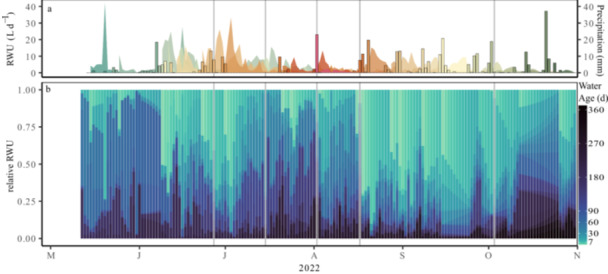
Absolute precipitation water uptake after each precipitation event based on root water uptake (RWU) isotopic composition weighted by sap flow and precipitation amount (bars) (a). Water age distributions in tree xylem water based on the modelled root water uptake (RWU) during the five defined periods in summer 2022. Darker colours depict an older water age, lighter colours a younger water age (b). [Color figure can be viewed at wileyonlinelibrary.com]

The results of the MixSIAR model also allowed the estimation of RWU sources throughout the vegetation period and water transit times of precipitation events. Our data clearly highlight the importance of winter and spring precipitation, but also precipitation events later in the season. Winter (2021/2022) and spring precipitation (2022) showed the longest transit times in tree xylem with an average of 206 ± 60 days compared to precipitation fallen later in the year. Especially in early summer, uptake of spring precipitation water was high due to high sap flow rates (Figure 5a), providing the main source (~ 80%) of the trees' sap flux (e.g., during 28 May 2022−02 June 2022).

Our results also showed a more rapid uptake of precipitation fallen during drought, indicated by high xylem water contributions. Therefore, transit times for summer precipitation were lower than of winter and spring precipitation (62 ± 11 days) and decreased further to 30 ± 8 days during dry down and the drought in July and August. During drought recovery, recent precipitation water from large events (e.g., 17 August 2022–20 August 2022, 39.1 mm) was the main source of tree xylem water with contributions of up to 77.3% ± 11.3%, highlighting the importance of large precipitation events in the growing season for tree water fluxes. While the transit times in xylem water were low for the first precipitation events directly after the drought (12 ± 0 days), larger precipitation events ( > 20 mm) were detectable in xylem water for more than 30 days. Precipitation fallen after September was still present in xylem water until the end of the measurement period at the end of October.

### 3.6 Significant Changes in Seasonal Water Ages of Tree Xylem Water

Xylem water ages changed markedly during the vegetation period. In May and the beginning of June, xylem water was mainly composed of water with an age of 30–60 days (Figure 5b) due to the high contribution of spring precipitation to RWU. Heavy precipitation events in June led to an increase in recent precipitation water in the xylem water and thereby an increase of younger water in tree xylem. Results indicate a direct use of recent precipitation marked in a younger xylem water age. Xylem water ages changed from recent precipitation during wetter periods to an uptake of older winter and spring precipitation during drought. The cumulative age distribution analysis (Supporting Information S1: Figure S1) showed that during drought 77% of the xylem water age was significantly higher (*p* < 0.05) compared to the preceding wet period. This was due to the lack of precipitation particular visible in July. Drought recovery was initiated by precipitation in mid‐August, which immediately contributed to more than 70% to RWU leading to a significantly younger xylem water composition (*p* < 0.05). Results confirm the swift response to recent precipitation as also seen in RWU depth dynamics.

### 3.7 Drought Effect on the Velocity and Percentage of Precipitation Water Uptake

**Figure 6:**
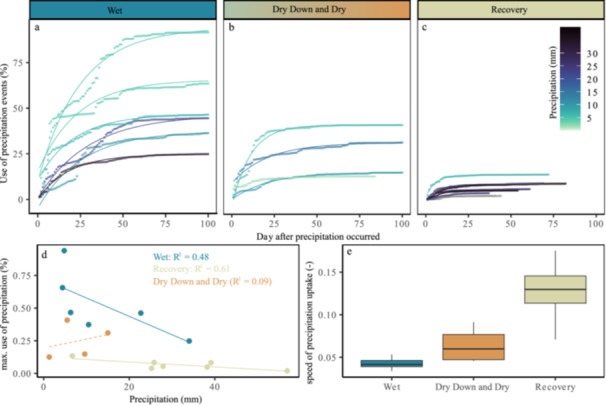
Precipitation water usage (%) followed an asymptotic regression during the initial wet period, drought and recovery period (a–c); the relationship of precipitation (mm) and the maximum percentage a tree can use per precipitation event for the initial wet and the recovery period (d). Too few precipitations occurred during drought to enable a calculation of the relationship (dashed orange line). Asterisk indicate significant differences in the proportional to the relative rate of precipitation use increase over time (i.e., the speed of precipitation water uptake) (e). [Color figure can be viewed at wileyonlinelibrary.com]

We further estimated the cumulative uptake of single precipitation events over the course of 2022, and found that the use of a precipitation water can be best described by an asymptotic regression (Figure 6a–c). The regression varied over the course of the season showing the velocity of precipitation water uptake to be lowest during the wet spring period. Interestingly the velocity of precipitation water uptake during the recovery period was significantly higher compared to the spring and drought period (both *p* < 0.01) (Figure 6e).

Furthermore, the maximum attainable percentage of water taken up from single precipitation events correlated with the amount of precipitation (mm) (Figure 6d). Water from smaller precipitation events were taken up to a larger percentage. Significantly less precipitation water was taken up during the recovery period than during the initial wet summer period (*p* < 0.01). Our results also illustrate that the use of precipitation water was affected by drought leading to a reduction of precipitation use during and after the drought albeit with a faster initial uptake. For example, more than 50% of precipitation events < 5 mm were taken up during the initial wet period while only < 25% of each precipitation event was taken up during the recovery period.

## 4 Discussion

To better assess the impact of changes in precipitation frequency and intensity on forest ecosystems, we aimed to gain a better understanding of changes in RWU and the importance of precipitation events to RWU under drought. Single precipitation events can provide important water input under drought, but little is known if and how long they events remain available to plants in the soil and contribute to RWU. Likewise, it is still under discussion if winter precipitation is the main source for tree water fluxes in the growing season of temperate forest ecosystems or to what extent precipitation events during the vegetation period can contribute significantly. This requires knowledge of the plasticity to adjust RWU depth in trees. Our main objective was therefore to investigate the RWU dynamics of *F. sylvatica* at a daily resolution. We focused on detecting potential changes in RWU depth and water ages. We investigated responses to single precipitation events before, during, and after a severe drought. High‐resolution water stable isotope data from a temperate forest ecosystem enabled us to detect seasonal changes in water transit times, precipitation‐dependent shifts in RWU depth and changes in precipitation usage throughout the course of the 2022 severe drought.

### 4.1 Significant Changes in RWU Depth and Water Ages During the Severe 2022 Drought

A shift of RWU to deeper soil layers can occur dynamically as a drought adaptation strategy as it has been described especially for arid ecosystems (e.g., Schwinning et al. 2002; Voltas et al. 2015; Carrière et al. 2020; Dubbert et al. 2019). By changing the depth of RWU to deeper soil layers with higher VWC, trees were able to maintain more positive water potentials and transpiration rates. Therefore, we hypothesised that *F. sylvatica* shifts its water uptake to deeper soil layers during drought, which could be confirmed. During the severe 2022 drought, we found dynamic changes of RWU to deeper soil layers compared to pre‐drought conditions. Those were associated with the use of winter and spring precipitation water. Thereby, shifts to deeper soil layers were more dynamically as expected and reversed immediately when precipitation events occurred. Changes to deeper soil layers occurred after several days without precipitation and gradual soil water depletion. Contrary, shifts of RWU to upper soil layers occurred within 2 to 4 days in response to single precipitation events. Our findings indicate a high plasticity in RWU depth, which is following the water availability in the soil, caused by a decreased hydraulic conductance in soil layers with reduced soil moisture (Carminati et al. 2013; Carminati and Javaux 2020; Javaux et al. 2008; Kühnhammer et al. 2020). Bachofen et al. (2024) evaluated 49 studies with regard to plant water uptake depth and concluded that woody plants take up water from soil layers with highest water availability, although studies focusing on patterns in temperate forest ecosystems were slightly underrepresented. Also, Nehemy et al. (2021) found a change to deeper and wetter soil layers during a lysimeter experiment with *Salix ciminalis* trees. These changes to deeper soil water resources usually aid in maintaining the hydraulic integrity of a tree, for example, by stabilising water potentials, but also to replenish stem water resources (Amin et al. 2020; Kühnhammer et al. 2023). Therefore, RWU depth and the accessibility of water in the soil are important for predicting drought vulnerability of mature forest trees (Kahmen et al. 2022).

Furthermore, our results highlight the dynamic response of *F. sylvatica* to take up water from top soil layers almost immediately after a precipitation event. Heavy precipitation events led to a significant increase in RWU from upper soil layers and an increase in sap flow rates within 2 to 4 days, which was especially evident after the δ^2^H labelling pulse in August 2022. The rapid response of trees to precipitation events suggests that trees do directly access and use mobile precipitation water (Kahmen et al. 2021; Kinzinger et al. 2023; Kühnhammer et al. 2023). In general, shallow soil layers are of great importance to the trees due to higher nutrient availability (Kreuzwieser and Gessler 2010), which results to the highest fine root density in top soil layers (Claus and George 2005; Bello et al. 2019; Meißner et al. 2012).

We further hypothesised that the age of water used for transpiration changed significantly due to the shift in RWU depth. Indeed, xylem water ages changed significantly during the vegetation period from recently fallen precipitation ( = younger water) during wetter periods to winter and spring precipitation taken up during summer drought ( = older water). However, xylem water was youngest during drought recovery, highlighting the importance of precipitation events in the recovery period. The observed increase in water ages under drier conditions agrees with an increase in RWU depth. However it could additionally be caused by the increased use of tree storage water when mobile and capillary soil water uptake was limited during the 2022 drought. While studies on the age of tree xylem water are very scarce, this is in line with available research pointing to the increasing use of older precipitation events when the upper soil layers dry out and shifts to deeper soil layers occur (e.g., Ellis et al. 2024 for *Pinus conorta*; Floriancic et al. 2024 for *F. sylvatica* and *P. abies*). Other studies highlight the importance of stem water storage. Barbeta et al. (2020) observed an isotopic offset between soil and xylem water in *F. sylvatica* saplings during an experimental drought treatment and hypothesised that it is caused by water isotope heterogeneities within the soil pore and stem tissues indicating water storage effects. Kühnhammer et al. (2023) showed that stem water storage is increasingly used to sustain sap flow and transpiration in some tropical species with increasing drought. Knighton et al. (2020) also highlighted that RWU dynamics should take the use of tree storage water into account, particularly during periods of low transpiration. We found secondary enrichments in δ^2^H in xylem water during precipitation weeks after the labelling, which also potentially hint at the use of stem water storage for transpiration. Fresh precipitation in the post‐drought might have increased the available soil water, increasing sap flow and therefore also the use of stored, potentially labelled stem water resources (which were refilled during the ^2^H labelling). High stem water capacitance can increase the drought resilience of temperate tree species (Leuschner et al. 2024; Zweifel et al. 2001). However, its general role in tree drought resilience still requires further investigation. Alternatively, fresh precipitation might have washed out labelled water that filled fine pores in the dry soil after the pulse labelling and thereby making “older” labelled water available to the trees. For a better understanding of this temporal offset of labelled water use further measurements and analysis of soil water dynamics and stem water storage are required.

### 4.2 Influence of Drought on Precipitation Water Uptake

A daily analysis after irrigation showed a faster but proportionally smaller uptake of labelled water during drought compared to the pulse labelling in moister conditions. Similarly, during the recovery period, we found a significantly faster uptake of precipitation water during the 2022 drought compared to pre‐drought conditions. In general, smaller precipitation events remaining in the upper soil layers were taken up to a higher percentage than larger events. However, the fraction of single precipitation events, which was taken up by the trees, was significantly lower during the recovery period compared to predrought conditions. This limitation in absolute water uptake rates could hint at legacy effects of the 2022 severe drought (cf. Müller and Bahn 2022), potentially due to a high fine root mortality in the topsoil layers during drought (e.g., Mainiero and Kazda 2006). Additionally, sap flow rates in the recovery period did not reach pre‐drought values, despite replenished soil VWC and similar VPD. However, it is still not fully understood how drought legacies may affect RWU patterns of trees. As annual precipitation sums and precipitation variability of precipitations size drive RWU depth pattern by favouring plants with deeper rooting systems in environments with variable precipitation (Bachofen et al. 2024), changes in those patterns may cause species dependent drought legacy effects. Furthermore, reduced sap flow rates may also be related to an earlier leaf senescence caused by the severe drought (Peng et al. 2019; Frei et al. 2022).

### 4.3 Water Transit Times

Although the highest proportion of precipitation water was estimated to be taken up shortly after its occurrence (seen also in the uptake of labelled water after irrigation), our data also showed that stronger precipitation events can affect isotopic values in tree xylem water and soil profiles for weeks. This indicates long transit times of precipitation water in our trees of > 6 months especially of winter and spring precipitation, which is in line with other studies. For example, Gazis and Feng (2004) found transit times for deep soil water to be at least 4.5 months at different mixed forested sites. Also, Kahmen et al. (2021) found labelled irrigation until the end of an 81day experiment in the investigated tree species, including *F. sylvatica*. Similar transit times with > 55 days of the labelling pulse in the measured tree xylem were found by Werner et al. (2021) and Kühnhammer et al. (2023) for several tropical tree species at Biosphere 2 Tropical Rainforest and Magh et al. (2020) for *Abies alba* and *F. sylvatica* in a temperate forest ecosystem.

We further found that transit times varied seasonally, indicating that precipitation fallen in summer before the drought had much shorter transit times compared to winter and spring precipitation, similar to Benettin et al. (2019) in vegetated lysimeter experiment on *Salix viminalis*. The results highlight the importance of winter and spring precipitation for trees during the vegetation period(Martin et al. 2018; Scharnweber et al. 2020). Further understanding of source water usage and water transit times in trees can help to understand how precipitation can drive drought recovery processes in the future and how drought legacy effects may inhibit precipitation water uptake. Further research should consider species‐specific responses to stressors such as drought (Antunes et al. 2018; Dubbert & Werner 2018; Dubbert et al. 2019; Grossiord et al. 2017).

### 4.4 Model Evaluation

Using a Bayesian MixSIAR model, we were able to track the precipitation water uptake in the xylem of *F. sylvatica*. The model output showed the contribution of each precipitation event in percentage to the composition of RWU per day. The model uncertainty indicated that estimated RWU composition from natural abundance precipitation is prone to a higher uncertainty than using labelled water. Still, precipitation uptake pattern for the pulse labelling experiments and the natural abundance precipitation were similar, with for example, faster uptake but relative smaller use of labelled water/precipitation during drought compared to the wet period labelling/precipitation, Therefore, we argue that the model performance with natural abundance precipitation is sufficient to resolve changes in RWU in our system. This is in contrast to findings by von Freyberg et al. (2020), who concluded that water with natural isotopic abundance does not vary sufficiently for a clear source water distinction.

The relative depletion of xylem δ^2^H values in previous work has led to speculation on isotope fractionation in the unsaturated zone or within plant tissues, which could influence the results of mixing models (Barbeta et al. 2020; Ellsworth and Sternberg 2015; von Freyberg et al. 2020). Further uncertainties may arise from spatial heterogeneity of flow pathways through trees, for example, due to different root or crown connections (Schulte 2003; von Freyberg et al. 2020). Nevertheless, by analysing the water source using water from each precipitation event, we were able to estimate time lags and transit times of RWU, which are currently insufficiently quantified (von Freyberg et al. 2020).

## 5 Conclusions

In summary, continuous in‐situ measurements of water stable isotope revealed drought‐induced changes in RWU depth, water ages and water transit times of *F. sylvatica*. Trees showed a high temporal plasticity in RWU depth and swiftly responded to changes in soil water availability. RWU depth shifted to deeper soil layers after consecutive days without precipitation but immediately returned to top soil layers after precipitation events infiltrated the soil. Furthermore, we demonstrated the importance of winter precipitation during drought, but also growing season precipitation for tree water fluxes during moisture conditions and drought recovery. Changes in annual precipitation patterns under progressing climate change in the future may strongly impact drought resistance and recovery. Here, we found that the drought in 2022 already affected the uptake of precipitation water during the recovery period. Faster uptake albeit smaller percentage of precipitation water use after drought may be related to drought legacy effects causing sap flow rates to remain low despite replenished soil water sources and favourable atmospheric conditions. Our study revealed high plasticity in adjustments of water source and RWU depth within days, driven by changes in precipitation amount and soil water availability. However, these dynamics were markedly influenced by drought and should be considered when projecting trees' drought resilience under climate change.

## Conflicts of Interest

The authors declare no conflicts of interest.

## Supporting information

SupplementaryMaterial fff.

## Data Availability

The data that support the findings of this study are available from the corresponding author upon reasonable request.
