# Supplementary material for: Continuous In‐Situ Water Stable Isotopes Reveal Rapid Changes in Root Water Uptake by Fagus sylvatica During Severe Drought
Source: Plant Cell Environ. 2025 Jul 10;48(10):7627–39. doi: 10.1111/pce.70055 (PMC12415413; doi:10.1111/pce.70055)
Supplement: Supplementary file 1 — SupplementaryMaterial fff. [file PCE-48-7627-s001.docx]

**Supporting Information**


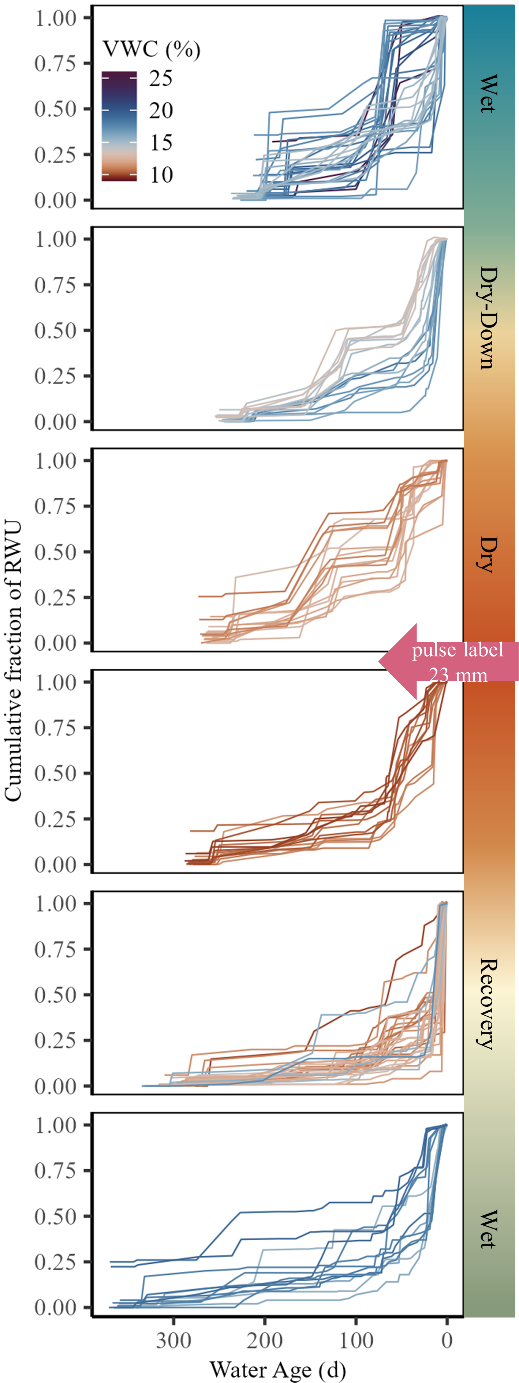


Figure S1: Cumulative distribution curves describing the age distributions in tree xylem water during the different phases.
